# Patient-specific risk factors contributing to blood culture contamination

**DOI:** 10.1017/ash.2022.22

**Published:** 2022-03-18

**Authors:** Sidra Liaquat, Lorena Baccaglini, Gleb Haynatzki, Sharon J. Medcalf, Mark E. Rupp

**Affiliations:** 1 Department of Epidemiology, College of Public Health, University of Nebraska Medical Center, Omaha, Nebraska; 2 Department of Biostatistics, College of Public Health, University of Nebraska Medical Center, Omaha, Nebraska; 3 Division of Infectious Diseases, University of Nebraska Medical Center, Omaha, Nebraska

## Abstract

**Objective:**

Contaminated blood cultures result in extended hospital stays and unnecessary antibiotic therapy. Patient-specific factors associated with blood culture contamination remain largely unexplored. Identifying patients at higher risk of blood culture contamination could alert healthcare providers to take extra precautionary measures to limit contamination in these patients, and thereby prevent associated adverse outcomes. We sought to identify patient-related factors that contribute to blood culture contamination in hospitalized patients.

**Design and setting:**

We conducted a secondary data analysis of a retrospective cohort study at an academic medical center.

**Patients:**

Study participants included 19,255 adult patients who had blood culture(s) performed during a hospital admission between June 2014 and December 2016.

**Methods:**

Data were analyzed to evaluate risk factors for blood culture contamination using logistic regression.

**Results:**

Among adult patients, we identified 464 contaminated episodes and 11,010 negative blood-culture episodes. Chronic obstructive pulmonary disease (adjusted odds ratio [AOR], 1.67; 95% confidence interval [CI], 1.20–2.34) and stay in an intensive care unit (ICU) during an admission (AOR, 1.41; 95% CI, 1.14–1.74) were associated with blood culture contamination. Other risk factors included race, body mass index, and admission from the emergency department. Subgroup analyses of patients admitted from the emergency department showed similar results.

**Conclusions:**

We identified patient-specific factors that increase the odds of false-positive blood cultures. By introducing mitigation strategies to limit contamination in patients with these risk factors, it may be possible to reduce the adverse clinical impact of blood culture contamination.

In the United States, 0.6%–6% of all blood cultures are contaminated with skin-residing organisms resulting in increased hospital stay and unnecessary antibiotic therapy, and emergence of antibiotic resistance.^1–4^ Blood culture contamination generally occurs prior to specimen processing in the laboratory during blood specimen collection and specimen handling.^5,6^ During venipuncture, bacteria on skin fragments can dislodge into the specimen.^7^ Using preventive measures during blood specimen collection and handling, such as antiseptic skin preparation, following appropriate venipuncture protocols, cleaning culture-bottle tops, utilizing sterile gloves and blood-culture collection kits, specimen diversion devices, double-needle technique, and having a dedicated phlebotomist team have been found to decrease contamination of blood cultures.^8–13^ Some studies have also shown that educating staff members on these preventive measures can help reduce blood culture contamination.^14^

In addition to specimen collection and handling, patient-specific factors can also contribute to blood culture contamination, including age, body mass index (BMI), comorbidities, and clinical status. Previous studies have shown that, compared to patients with negative blood cultures, emergency department (ED) patients with contaminated blood cultures were more likely to be older; to be of black race; to have higher BMI; to have end-stage renal disease, chronic obstructive pulmonary disease (COPD), or paralysis; and to be in a critical condition (triage level I and II) or septic shock.^15,16^ Mental status of the patient and the severity of underlying disease were risk factors for contaminated culture in hospitalized patients.^17^ To date, only limited studies have systematically explored patient-related factors in hospitalized adult patients that might contribute to blood culture contamination.

Contaminated blood cultures can be difficult to interpret and can have a negative impact on patient management by incurring treatment delays, exposing patients to unnecessary and inappropriate antibiotics, unnecessarily extending hospital stay, and requiring additional testing and consultation.^4,16,18^ In this study, we sought to determine patient-specific factors contributing to blood culture contamination in hospitalized patients.

## Methods

### Study design

This retrospective cohort study involved analyses of data stemming from patients with a hospital admission that included blood culture testing. All patients who had blood culture(s) performed at any time during an admission were included in the study population. This study was reviewed and approved by the University of Nebraska Medical Center (UNMC) Institutional Review Board.

### Study setting

Electronic medical record data from adult inpatients aged 18 years or older admitted between June 1, 2014, and December 31, 2016, at the University of Nebraska Medical Center were included in the study. Admissions that did not fit the study definitions of contaminated or negative blood culture episodes were excluded. To avoid bias, if a patient had >1 eligible admission, only the first admission was included in the analyses. Patients discharged from the emergency department were excluded; however, patients who were initially treated in the emergency department and subsequently admitted to the hospital were included. A detailed description of the study participants has been reported elsewhere.^4^

### Study definitions

#### Contaminated blood culture

Blood cultures were continuously monitored via an automated system (Bactec; Becton Dickinson, Franklin Lakes, NJ) with positive specimens being further analyzed for isolate identification and antimicrobial susceptibility via a FilmArray rapid identification system (BioFire Diagnostics, Salt Lake City, UT) and a semi-automated susceptibility system (Microscan; Siemens, Munich, Germany) with further testing via matrix-assisted laser desorption/ionization-time of flight (MALDI-TOF; Bruker Sensityper, Bruker, Billerica, MA) as needed. A blood culture was considered contaminated if skin-residing organism(s) were identified in 1 of the 2 or more blood-culture sets. Skin-residing organisms included coagulase-negative staphylococci (CoNS), *Cutibacterium acnes* (formerly known as *Propionibacterium acnes*), *Micrococcus* spp, viridians group streptococci (VGS), *Corynebacterium* spp, and *Bacillus* spp.

#### Contaminated episode

A patient admission was categorized as a contaminated episode if the first ordered blood culture was reported as contaminated (based on the definition of contaminated blood culture) and any subsequent blood culture during that same admission was negative.

#### Negative episode

A patient admission was categorized as a negative episode if all blood cultures in that admission were negative for any organism.

#### Positive episode

If 1 or more positive blood cultures were reported with organisms other than likely contaminants (listed above), the admission was categorized as a positive episode. Polymicrobial blood cultures in which a likely contaminant was recovered in combination with a likely true pathogen were regarded as positive episodes.

#### Equivocal episode

Any combination of blood culture results during a patient admission that did not fall under contaminated, negative, or positive episode were labelled as an equivocal episode.

### Outcome

The study outcome was blood culture episode categorized as contaminated or negative based on blood culture results obtained during the admission. We considered analyzing patients with contaminated blood cultures and those with negative blood cultures to be the optimal comparative approach to determine risk factors for contamination. Inclusion of patients with positive blood cultures or those with equivocal results risked introduction of a variety of confounding variables associated with true bacteremia.

### Variables

Exposure variables included age, which was categorized based on quartiles (<50 years, 50–61 years, 62–73 years, or >73 years), sex (male or female), race (white, black, or other), BMI (kg/m^2^), smoking status (smoker/former smoker/current smoker, or non-smoker), alcohol status (drinks alcohol/drinks alcohol daily/drinks alcohol occasionally, or does not drink alcohol), and medical insurance (insured or uninsured). In addition to end-stage renal disease (ESRD), which has been previously implicated in increased blood culture contamination, we also included COPD, liver cirrhosis, and diabetes mellitus in the analysis. These comorbidities were extracted from *International Classification of Disease, Tenth Revision* (ICD-10) codes listed in the electronic medical record of each admission. Additional variables included stay in intensive care unit (ICU) during admission, admission from the emergency department, and anatomic location of blood drawn for blood culture (central intravenous catheter or peripheral vein). In general, blood cultures obtained from the peripheral vein were drawn by trained phlebotomists, and blood cultures from central venous catheters were drawn by nurses. For quality control purposes, a convenience subset of 50 patient admission records were cross checked manually.

### Power calculation

A sample of 11,010 negative blood culture episodes and 464 contaminated episodes provided 80% power to detect a standardized mean difference of 0.132. G*Power software was used for the power analysis.^19^

### Statistical analysis

For descriptive statistics, means and standard deviations were calculated for continuous variables, and counts and percentages were calculated for categorical variables. We used χ^2^ and 2-sample independent *t* tests to determine associations between the outcome and the covariates. Covariates associated with the outcome variable in crude analyses at α = 0.1 were included in a multivariable logistic regression model. Age and sex were maintained in the final model a priori. A forward stepwise selection at α = .05 was utilized to create the final model, and the Akaike information criterion (AIC) value was used to assess model fit. Results were reported as crude odds ratios (ORs) and adjusted odds ratios (AORs) with 95% confidence intervals. All analyses were conducted using SAS version 9.4 software (SAS Institute, Cary, NC).

## Results

We identified 19,255 admissions between June 1, 2014, and December 31, 2016. Variables such as race, marital status, health insurance and BMI had <5% missing data. Up to 10% of the data were missing for smoking status, and up to 30% were missing data for alcohol status. Complete data were available on all other variables.

We applied the following exclusion criteria: patient <18 years of age (n = 1,419), positive/true bacteremia (n = 1,395), equivocal results (n = 431), and repeat hospital admissions (n = 4,506). After exclusions, the final analytical sample consisted of 11,474 patient admissions with 11,010 negative and 464 contaminated episodes (Table 1). Overall, the 2 groups had comparable clinical and sociodemographic characteristics. Nearly half of the patients in both groups were male, and 80% were white race. More patients in the contaminated group (10%) had COPD versus patients with negative episodes (6%). Likewise, 35% of patients with contaminated blood-culture episodes included ICU stay at some point during the hospitalization, compared to 28% of patients with negative blood-culture episodes.

**Table 1.** Patients’ Clinical and Sociodemographic Characteristics by Blood Culture Status

Variables	Blood Culture Episode		
	Negative (N = 11,010), No. (%)	Contaminated (N = 464), No. (%)	Overall (N = 11,474, No. (%)
**Age**			
<50 y	2,916 (26.4)	108 (23.3)	3,024 (26.4)
50–61 y	2,659 (24.2)	128 (27.6)	2,787 (24.3)
62–73 y	2,727 (24.8)	112 (24.1)	2,839 (24.7)
>73 y	2,708 (24.6)	116 (25.0)	2,824 (24.6)
**Sex**			
Male	5,730 (52.0)	236 (50.9)	5,966 (52)
Female	5,280 (48.0)	228 (49.1)	5,508 (48)
**Race^a^**			
Black	1,106 (10.1)	61 (13.2)	1167 (9.6)
White	9,058 (82.7)	376 (81.6)	9,434 (82.2)
Other^b^	791 (7.2)	24 (5.2)	815 (7.1)
**Body mass index (kg/m** ^2^ **)^c^**			
Mean (SD)	29.5 (8.5)	30.6 (10.1)	
**Admission from ED**			
Yes	6,865 (62.4)	314 (67.7)	7,129 (62.1)
No	4,145 (37.6)	150 (32.3)	4295 (37.4)
**ICU stay**			
Yes	3,030 (27.5)	164 (35.3)	3,194 (27.8)
No	7,980 (72.5)	300 (64.7)	8,280 (72.2)
**Health insurance^a^**			
Yes	10,062 (92.1)	429 (92.7)	10,491 (91.4)
No	866 (7.9)	34 (7.3)	900 (7.8)
**Location of blood draw^c^**			
Central intravenous (IV) catheter	1,081 (10.2)	43 (9.6)	124 (9.8)
Peripheral vein	9,529 (89.8)	406 (90.4)	9,935 (86.6)
**Alcohol status^d^**			
Drinks alcohol^e^	2,573 (32.8)	88 (26.5)	2,661 (23.2)
Does not drink alcohol	5,280 (67.2)	244 (73.5)	5,524 (48.1)
**Smoking status^f^**			
Smoker^g^	5,941 (59.4)	273 (64.5)	6,214 (54.2)
Nonsmoker	4,058 (40.6)	150 (35.5)	4,208 (36.7)
**Underlying disease**			
**Chronic obstructive pulmonary disease (COPD)**			
Yes	662 (6.0)	47 (10.1)	709 (6.2)
No	10,348 (94.0)	417 (89.9)	10,765 (93.8)
**Chronic kidney disease**			
Yes	9 (0.1)	1 (0.2)	10 (0.08)
No	11,001 (99.9)	463 (99.8)	11,464 (99.9)
**Liver cirrhosis**			
Yes	146 (1.3)	4 (0.9)	150 (1.3)
No	10,864 (98.7)	460 (99.1)	11,324 (98.7)
**Diabetes mellitus**			
Yes	1,178 (10.7)	60 (12.9)	1,238 (10.8)
No	9,832 (89.3)	404 (87.1)	10,236 (89.2)

Note: ED, emergency department; ICU, intensive care unit; SD, standard deviation.

a <1% missing data.

b Other includes Asian, Hawaiian, Pacific Islander, Native American.

c <5% missing data.

d <30% missing data.

e Drinks alcohol includes drinks alcohol daily, drinks alcohol every other day, drinks alcohol occasionally.

f <10% missing data.

g Smoker includes current smoker, former smoker, daily smoker.

In the multivariable analysis (Table 2), the adjusted odds of blood culture contamination were higher for patients with an ICU stay versus no ICU stay during their hospitalization (AOR, 1.41; 95% CI, 1.14–1.74). Patients with COPD had 1.67 higher adjusted odds of having a contaminated blood culture (95% CI, 1.20–2.34). Additionally, the adjusted odds of a contaminated blood culture result increased by 1.01 for every additional unit of BMI (AOR, 1.01; 95% CI, 1.00–1.02). Blacks had 1.35 higher adjusted odds of having a contaminated blood culture compared to whites (AOR, 1.32; 95% CI, 1.00–1.81). Patients admitted from the ED had 1.20 higher adjusted odds of having contaminated blood cultures than those not admitted from ED (AOR, 1.20; 95% CI, 0.96–1.50).

**Table 2.** Crude and Adjusted Odds Ratio for Blood Culture Contamination

Variables	Crude OR (95% CI)	Adjusted OR (95% CI)^a^
**Age group**		
<50 y	1	1
50–61	1.30 (1.00–1.69)	1.22 (0.92–1.61)
62–73 y	1.11 (0.85–1.45)	1.02 (0.76–1.37)
>73 y	1.16 (0.88–1.51)	1.00 (0.74–1.35)
**Sex**		
Male	1	1
Female	1.05 (0.87–1.26)	1.09 (0.89–1.34)
**Race**		
White	1	1
Black	1.33 (1.01–1.75)	1.35 (1.00–1.81)
Other^b^	0.73 (0.48–1.11)	0.83 (0.54–1.30)
**ICU stay**		
No	1	1
Yes	1.44 (1.18–1.75)	1.41 (1.14–1.74)
**COPD**		
No	1	1
Yes	1.76 (1.29–2.41)	1.67 (1.20–2.34)
**Admission from ED**		
No	1	1
Yes	1.26 (1.04–1.54)	1.20 (0.96–1.50)
**Body mass index (kg/m** ^2^ **)**	1.01 (1.00–1.03)	1.01 (1.00–1.02)
**Smoking status**		
No	1	1
Yes	1.24 (1.02–1.52)	1.18 (0.95–1.47)

Note: OR, odds ratio; CI, confidence interval; COPD, chronic obstructive pulmonary disease; ED, emergency department; ICU, intensive care unit.

a Adjusted for age, sex, race, body mass index, smoking status, presence of COPD, admission from ED, and ICU stay.

b Others includes Asian, Hawaiian, Pacific Islander, Native American.

A subgroup analysis of patients admitted to the hospital from ED showed similar results. The odds of contamination were higher in patients who were Black (AOR, 1.53; 95% CI, 1.12–2.09), who had COPD (AOR, 1.84; 95% CI, 1.26–2.68), who had a higher BMI (AOR, 1.02; 95% CI, 1.0–1.03), and whose admission included an ICU stay (AOR, 1.58; 95% CI, 1.23–2.03).

## Discussion

The results of this study showed that patients admitted from the emergency department were more likely to have contaminated blood cultures. Blood cultures drawn in emergency departments may be more susceptible to contamination due to staff turnover, the need to collect cultures in critically ill patients during resuscitation, and the time pressure of obtaining cultures before the first dose of antibiotics.^15,20,21^

Patients in a critical condition had higher odds of having contaminated blood cultures, and this has been observed in other studies.^15–17^ We used ICU stay during admission as a proxy for severity of clinical status and observed higher odds of blood contamination in patients with ICU stay. The higher odds of blood culture contamination in the ED or ICU could apply to critically ill patients, who often tend to be hypovolemic or hypotensive. We speculate that this condition may lead to multiple needle sticks to draw blood from less prominent and fragile veins resulting in a contaminated blood culture.

We observed a significant association between COPD and contamination of blood cultures (AOR, 1.67; 95% CI, 1.20–2.34) as was also noted in the study by Klucher et al.^17^ A possible explanation for this association can be explained by the link between COPD and peripheral vascular disease (PVD), as demonstrated by other studies.^22^ Patients with PVD have thin and weak peripheral vasculature, which could result in multiple needle sticks to draw blood. As a result, patients with COPD might be more prone to blood culture contamination.

Chang et al^15^ found old age and end-stage renal disease (ESRD) to be significant risk factors for blood culture contamination. We did not find a strong association between age and contaminated blood cultures. Additionally, we could not evaluate the association between ESRD and blood culture contamination, since we did not have a sufficient number of participants with ESRD.

Similar to Klucher et al,^17^ we found that Blacks had higher odds of having a contaminated blood culture than Whites (AOR, 1.35; 95% CI, 1.00–1.81). There is no known biological correlation to suggest a higher tendency of blood culture contamination based on racial or ethnic background. However, this finding might be another reflection of health and socioeconomic disparities that exist in our society.^23^ For example, Blacks are more likely to develop early-onset COPD, which puts them at higher odds of blood culture contamination.^17,24^ Additionally, we observed an association between BMI and blood culture contamination with a higher odds of contamination for every additional unit of BMI (AOR, 1.01; 95% CI, 1.00–1.02), which was also demonstrated by Klucher et al.^17^ With regard to the increased risk of blood culture contamination based on race, Blacks have a higher rate of obesity than non-Hispanic Whites.^25^ In patients with higher BMI, and specifically those who are obese (BMI 30 to <40) or morbidly obese (BMI ≥40), it might be hard to find veins due to excess adiposity leading to several needle sticks, resulting in blood culture contamination.

This study had several limitations. It was a retrospective review of electronic medical records; thus, it was not feasible to verify the accuracy of archived patient data. Being a historical cohort, it was not possible to control for all possible confounders. Additionally, for patient admissions that included ICU stay, we were not able to ascertain whether the first blood culture was drawn during an ICU stay or at any other time during the hospitalization. Although ICU stay and admission from the emergency department may have served as surrogate markers for severity of illness, more direct measures of severity of illness and comorbid conditions such as the Pitt bacteremia score, APACHE II score, or Charlson comorbidity index were not collected or calculated.

Approximately 30 million blood cultures are performed in the United States each year, with most hospitals experiencing contamination rates of ∼2%–3%. Blood culture contamination results in substantial adverse effects such as prolonged hospital stay and inappropriate antibiotic use. Unfortunately, our understanding of patient-related factors potentially contributing to contamination of blood cultures, particularly in hospitalized patients is incomplete. Knowledge of these contributing factors can help hospital epidemiologists, antimicrobial stewardship personnel, clinical microbiologists, and bedside clinicians to identify patients at higher risk and to introduce additional measures to prevent contamination. For example, patients with conditions known to be associated with blood culture contamination (eg, COPD, elevated BMI, etc) could have an electronic alert directed to phlebotomists when blood cultures are ordered, notifying them of the increased risk and triggering additional measures to ensure aseptic technique or use of devices to limit blood culture contamination (eg, initial specimen diversion device). Early identification of patients at greater risk of blood culture contamination can assist clinicians in providing better care for these patients. Similar to predictive models assisting clinicians with the recognition of potentially bacteremic patients,^26^ knowledge of patient-specific factors associated with contamination could help in formulating predictive equations to better identify patients at higher risk of blood culture contamination.
